# The Effect of Food on the Single‐Dose Bioavailability and Tolerability of the Highest Marketed Strength of Duloxetine

**DOI:** 10.1002/cpdd.759

**Published:** 2019-12-02

**Authors:** Simona Rizea‐Savu, Simona Nicoleta Duna, Adrian Ghita, Adriana Iordachescu, Marinela Chirila

**Affiliations:** ^1^ 3S‐Pharmacological Consultation & Research GmbH Harpstedt Germany; ^2^ 3S‐Pharmacological Consultation & Res. SRL Bucharest Romania; ^3^ Pharma Serv International SRL Bucharest Romania; ^4^ Medochemie Ltd Limassol Cyprus

**Keywords:** duloxetine, bioequivalence, bioavailability, pharmacokinetics, tolerability, HPLC‐MS/MS

## Abstract

Duloxetine is a combined serotonin and norepinephrine reuptake inhibitor indicated in adults for the treatment of major depressive disorder, diabetic peripheral neuropathic pain, and generalized anxiety disorder. The aim of these studies was to evaluate the effect of food on the pharmacokinetics and safety of duloxetine 60‐mg gastroresistant hard capsules following single‐dose administration. The data were obtained from 2 phase 1 bioequivalence studies, 1 in a fasting state and the other under fed conditions. Both studies have shown that, when administered as a single dose in the same prandial state, the test and reference duloxetine treatments were bioequivalent and exhibited similar safety profiles. The mean fed and fasting pharmacokinetic parameters and drug‐related adverse events from the 2 studies were compared in order to assess the effect of food on the duloxetine bioavailability and respectively, tolerability. Administration of duloxetine in fed conditions increased peak plasma concentration by up to 30% and delayed mean time to peak concentration by an average of 1.15 hours while having an insignificant effect on extent of absorption (area under the plasma concentration–time curve in fed state within ±6% as compared with fasting conditions). Even though peak plasma levels were substantially higher in the fed state, there was no negative impact on the drug's safety profile. Actually, administration with food resulted in a lower average number of adverse events per single dose exposure. The negligible variation in overall systemic exposure suggests that efficacy remains unchanged irrespective of administration conditions; however, a better tolerability of the 60‐mg dose is expected when the drug is taken with food.

Duloxetine is a combined serotonin and norepinephrine reuptake inhibitor that weakly inhibits dopamine reuptake with no significant affinity for histaminergic, dopaminergic, cholinergic, and adrenergic receptors.^1^ It works by preventing the 5‐hydroxytryptamine and norepinephrine neurotransmitters from being taken back up into nerve cells in the brain and spinal cord, thus increasing their concentration in the spaces between nerve cells and enhancing the level of communication between cells. Because these neurotransmitters are involved in maintaining good mood and reducing the sensation of pain, blocking their reuptake into nerve cells improves the symptoms of depression, anxiety, and neuropathic pain.^2^ Due to its pharmacodynamic effects, confirmed in several placebo‐controlled clinical trials,^3^, ^4^, ^5^ duloxetine is indicated in adults for the treatment of major depressive disorder, diabetic peripheral neuropathic, pain, and generalized anxiety disorder.

Duloxetine hydrochloride is an acid‐labile substance suitable for pharmaceutical development only in enteric‐coated dosage forms that prevent degradation of the active substance in the acidic environment of the stomach.^6^

Duloxetine is well absorbed after oral administration. There is an apparent slight nonlinear behavior with a more than proportional increase in plasma concentration across the duloxetine dosage range of 40 mg/d to 120 mg/d.

Following [^14^C]‐duloxetine oral administration via enteric‐coated tablet, peak plasma concentration (C_max_) is reached at a median of 6 hours for duloxetine and also for total radioactivity. Duloxetine accounts for less than 3% of the circulating radioactivity based on mean area under the curve (AUC_0‐t_) values, due to numerous metabolites with elimination half‐lives substantially longer than that of duloxetine. Mean total recovery of radioactivity after 312 hours is about 90.5% with 72.0% excreted in the urine, mainly as conjugated metabolites. The major metabolites found in plasma are glucuronide conjugates of 4‐hydroxy duloxetine (M6), 6‐hydroxy‐5‐methoxy duloxetine (M10), and 4,6‐dihydroxy duloxetine (M9), and a sulfate conjugate of 5‐hydroxy‐6‐methoxy duloxetine (M7).^7^

Demographic and physiological characteristics found to influence the pharmacokinetics of duloxetine include sex, smoking status, age, ethnicity, cytochrome P450 (CYP) 2D6 genotype, hepatic function, and renal function. Of these, only impaired hepatic function or severely impaired renal function warrant specific warnings or dose recommendations. Interaction studies show that CYP1A2 inhibition increases duloxetine exposure to a clinically significant degree (AUC_0‐t_ increased by 460% in the presence of fluvoxamine),^8^ whereas the exposure of duloxetine in the presence of CYP2D6 inhibitors or in CYP2D6 poor metabolizers is increased to a lesser extent (paroxetine, a potent CYP2D6 inhibitor, increased duloxetine exposure by only 60%, to a level similar to duloxetine exposure in CYP2D6 poor metabolizers).^9^ Apart from being a substrate of cytochrome CYP2D6, pharmacokinetic interaction studies have also shown that duloxetine is a moderately potent CYP2D6 inhibitor.^10^

The purpose of this article is to evaluate the effect of food on the pharmacokinetic parameters and safety of duloxetine 60‐mg gastroresistant hard capsules following single‐dose administration. The data were obtained from 2 phase 1 bioequivalence studies, 1 in the fasting state and the other under fed conditions. Both were designed as 2‐period, crossover, randomized studies in healthy white male and female volunteers. The aim of each study was to evaluate the bioequivalence of a generic formulation relative to the innovator product after oral administration of a single dose in fasting and fed conditions, respectively, and also to evaluate the safety of study treatments. The 2 studies shared remarkable similarities in terms of selected study population, clinical site, investigational staff, clinical and analytical data collection, and data‐processing techniques. The sampling and plasma handling techniques were identical, and the same analytical laboratory and the same validated high‐performance liquid chromatography–tandem mass spectrometry (HPLC‐MS/MS) methods were employed for analysis of the PK samples from both studies. The results were thereafter processed in the same manner using the same statistical methods.

The pharmacokinetic and safety data gathered from the 2 studies were therefore considered suitable for investigating the effect of food on the single‐dose bioavailability and tolerability of the highest marketed strength of duloxetine.

## Methods

### Protection of Human Subjects

The 2 phase 1 studies were conducted at the Clinical Center of 3S Pharmacological Consultation & Research SRL (located in Suceava county, Romania, EU), following unconditional approval from the National Bioethics Committee for Medicines and Medical Devices and the National Agency for Medicines and Medical Devices of Romania, EU. All subjects gave their written informed consent before they underwent any study‐related procedures and were free to withdraw from the trials at any time. Clinical investigations were conducted according to the Declaration of Helsinki principles and Good Clinical Practice.

### Investigational Products

The investigational products administered in the 2 studies were Cymbalta 60‐mg gastroresistant hard capsules (Eli Lilly Netherlands BV, Utrecht, the Netherlands) used as reference standard and Duloxetine Medochemie 60‐mg gastroresistant hard capsules (Medochemie Ltd, Limassol, Cyprus) used as test product. The same batches of test and reference were used in both studies, fasting and fed.

### Study Design and Subject Profile

Two phase 1 bioequivalence studies were conducted, 1 single‐dose study with drug administration under fasting conditions and 1 single‐dose study with drug administration under fed conditions. Both studies were open label and block randomized, having a 2‐period, 2‐sequence crossover design with a 7‐day washout between consecutive doses. The enrolled subjects were healthy, adult, male and female volunteers of white descent, with body mass index within 18.5 to 30.0 kg/m^2^ and who did not suffer from rare hereditary problems of glucose intolerance, the Lapp lactase deficiency, glucose‐galactose malabsorption, or gluten intolerance.

During the fasting study, a single dose of duloxetine as Medochemie 60‐mg gastroresistant hard capsules or Cymbalta 60‐mg gastroresistant hard capsules was administered per study period, orally, with 200 mL of still bottled water, after at least 8 hours of overnight fasting.

During the fed study, 1 single dose of 60 mg duloxetine was administered per study period (same batches of the same 2 formulations used in the fasting study), orally, with 200 mL of still bottled water, exactly 30 minutes after the subjects started consuming a high‐fat, high‐calorie breakfast meeting the nutritional breakdown recommended by the European Medicines Agency Committee for Medicinal Products for Human Use Guideline on the Investigation of Bioequivalence.

The pharmacokinetic parameters calculated in each study were AUC_0‐t_, C_max_, time to peak concentration (T_max_), AUC extrapolated to infinity (AUC_0‐∞_), half‐time of elimination, and mean residence time, and bioequivalence assessment was based on plasma drug levels of duloxetine.

The safety parameters analyzed in each study were the adverse events reported and the clinical and laboratory results from the screening and study exit examinations.

### Handling and Bioanalysis of Study Samples

#### Standards and Reagents

The reference standard duloxetine hydrochloride and the internal standard D3‐duloxetine oxalate were purchased from AlsaChim, Illkirch Graffenstaden, France. Ammonium acetate, ammonium formiate, acetic acid, dimethylsulfoxide, methanol, n‐hexane, *tert*‐butyl‐methyl ether, and water for chromatography were of analytical or HPLC grade, purchased from either Merck (Darmstadt, Germany) or Fluka (Leipzig, Germany).

#### Equipment

For the fasting study, analyses were carried out on MPX‐2 multiplexing HPLC systems (Applied Biosystems‐Sciex, Concord, Ontario, Canada) comprised of cooled CTC autosamplers (CTC Analytics, Zwingen, Switzerland) with 2 Rheodyne injection valves coupled with Schimadzu LC‐20AD pumps and Schimadzu DGU‐20A5 degassers (Shimadzu, Kyoto, Japan). The software MPX‐2 controlled all multiplexing functions of the high‐throughput HPLC system.

For the fed study, analyses were carried out on a Cohesive model Aria HPLC system (Cohesive Technologies, Franklin, Massachusetts) consisting of a cooled CTC autosampler (CTC Analytics, Zwingen, Switzerland), 2 Agilent series 1100 binary gradient pumps with Agilent degasser and 2 Agilent series 1100 quaternary gradient pumps with Agilent degasser (Agilent, Wilmington, Delaware). The Aria software (Cohesive Technologies, Franklin, Massachusetts) was used to control the 2 HPLC systems running in parallel on 2 separate columns (high‐throughput HPLC).

The mass spectrometers utilized were an AB‐Sciex model API 5000 (fasting study) and an AB‐Sciex API 5500QTRAP (fed study), both equipped with atmospheric pressure electrospray ionization interface (Turbo V) (AB Sciex, Framingham, Massachusetts).

Data were collected and processed using the Analyst software (Version 1.6 of AB Sciex, Foster City, California).

#### Liquid Chromatography and Mass Spectrometric Conditions

Except for the partial revalidation concerning changes of analytical equipment in the fasting study (different mass spectrometer), the same HPLC‐MS/MS conditions and sample preparation processes next described were used for duloxetine quantification in both studies. Chromatographic separations were carried out using reversed‐phase Supelco Discovery C18 (12.5 cm × 2.1 mm, 5 µm silica packing) analytical columns. The mobile phase consisted of methanol/ammonium acetate in water + acetic acid, premixed.

Samples of 5 µL were loaded onto the column, separated, and eluted in isocratic conditions. The autosampler temperature was thermostated at 10°C nominal, and quantitative data were acquired in positive‐ion mode using a multiple reaction monitoring method. The ion spray voltage and the source temperature were set at 5000 V and 500°C. Research‐grade nitrogen was used as curtain gas and collision gas. The resolutions for both Q1 and Q3 were set at unit.

A summary of the ion transitions, declustering potentials, collision energies, and collision cell exit potentials is presented in Table 1.

**Table 1:** Optimal Positive Ion Mass Spectrometric Conditions for Multiple Reaction Monitoring in the Fasting and Fed Studies

Analyte/IS Name	Ion Transition	Dwell Time (ms)	Declustering Potential (V)	Collision Energy eV	Collision Cell Exit Potential (V)
Duloxetine	298.091 → 154.200	150	50	7	12
D_3_‐duloxetine (IS)	301.130 → 157.200	150	50	9	10

IS indicates internal standard.

#### Calibration Curves and Quality Control Samples

The duloxetine hydrochloride stock solutions were prepared at 1.000 mg/mL duloxetine free‐base concentrations, and the stock solutions of internal standard were prepared at 1.000 mg/mL D_3_‐duloxetine concentrations. These solutions were stored at –20°C nominal. A series of working solutions for preparation of the 8‐point calibration curves, and the plasma quality control samples were obtained by mixing and diluting the stock solutions with pooled human plasma derived from blank blood samples collected on Li‐heparin from mixed‐sex healthy volunteers. Spiked quality control samples were prepared at 0.300, 8.000, 40.000, and 80.000 ng/mL, and the range of the calibration curves was 0.100 ng/mL (lower limit of quantification) to 100.000 ng/mL (upper limit of quantification).

#### Study Samples

For the quantification of duloxetine plasma levels, venous blood samples of 5 mL were drawn in tubes containing Li‐heparin as anticoagulant before dosing and at 1.0, 2.0, 3.0, 4.0, 5.0, 5.5, 6.0, 6.5, 7.0, 8.0, 9.0, 9.5, 10.0, 10.5, 11.0; 12.0, 14.0, 18.0, 24.0, 36.0, 48.0, and 72.0 hours after study drug administration in each period of the 2 studies. After collection, the blood samples were centrifuged under refrigeration (10 minutes at 1500*g* and a nominal temperature of 4°C). The samples were stored at –20°C or colder until submitted to analysis. Before analysis, plasma samples were thawed, mixed for 3 minutes, and centrifuged for 3 minutes at 2000*g* and 20°C nominal. Aliquots of samples were spiked with internal standard (D_3_‐duloxetine), extracted with n‐hexane/*tert*‐butyl‐methyl ether solution, vortexed, and centrifuged; supernatants were evaporated to dryness under air stream, reconstructed with a methanol/water solution, mixed, and centrifuged; finally, the samples were transferred in the autosampler to be injected. The analytical work was performed according to good laboratory practice principles and current European Medicines Agency requirements.^8^ The analytical method was fully validated before starting the analysis of study plasma samples. The method was verified for linearity, quantification limits, assay specificity, between‐run and within‐run precision and accuracy, analyte recovery, and stability in stock solution and biological matrix under processing conditions during the entire period of storage. The between‐run accuracy range was 100.61% to 103.86% in the fasting study and 99.43% to 101.65% in the fed study; the within‐run accuracy range was 98.44% to 105.03% in the fasting study and 97.75% to 100.98% in the fed study.

### Pharmacokinetic and Statistical Analyses, Including Evaluation of the Food Effect on Duloxetine Bioavailability

Noncompartmental PK analysis was performed using SAS statistical software (SAS Institute Inc, Cary, North Carolina). ANOVA was performed on natural logarithm–transformed C_max_, AUC_0‐t_, and AUC_0–∞_ using the General Linear Models procedure fitted in SAS software using the method of least squares. Descriptive statistics were performed for all pharmacokinetic parameters.

The mean fed and fasting pharmacokinetic profiles were compared in order to assess the effect of food on the bioavailability of duloxetine.

### Statistical Analyses on Adverse Events and Evaluation of the Food Effect on Duloxetine Safety

In each study a single sample proportion test was applied by treatment group (test versus reference) for the incidence of subjects having encountered adverse events and the incidence of adverse events (run in NCSS software, version 07.1.21; NCSS LLC, Kaysville, Utah). The limit of significance was set at 0.05.

## Results

### Study Subjects and Pharmacokinetic Results

#### Demographic Data and Body Metrics of the Population Enrolled

A total of 72 healthy male and female volunteers were enrolled in the fasting study, of whom 66 subjects completed the clinical part of the trial and were included in the per‐protocol PK population. Of the enrolled subjects, 71 received at least 1 dose of study medication and were included in the safety population.

In the fed study a total of 44 healthy male and female volunteers were enrolled, of whom 42 subjects completed the clinical part of the trial and were included in the per‐protocol PK population. All enrolled subjects received at least 1 dose of study medication and were included in the safety population.

The enrolled subjects were healthy white adults with the mean demographics and body metrics presented in Table 2.

**Table 2:** Mean Demographic Data and Body Metrics of the Population Enrolled in the 2 Studies

Study	Age (y)	Weight (kg)	Height (cm)	BMI (kg/m^2^)
	Mean (Range)	Mean (Range)	Mean (Range)	Mean (Range)
Fasting study	34.69	70.00	169.14	24.40
(N = 72)	(18‐57)	(50‐105)	(150‐190)	(19.0‐29.9)
Fed study	34.68	74.11	169.86	25.64
(N = 44)	(18‐53)	(53‐96)	(153‐186)	(18.6‐29.8)

BMI indicates body mass index.

#### Pharmacokinetic Results

**Figure 1:**
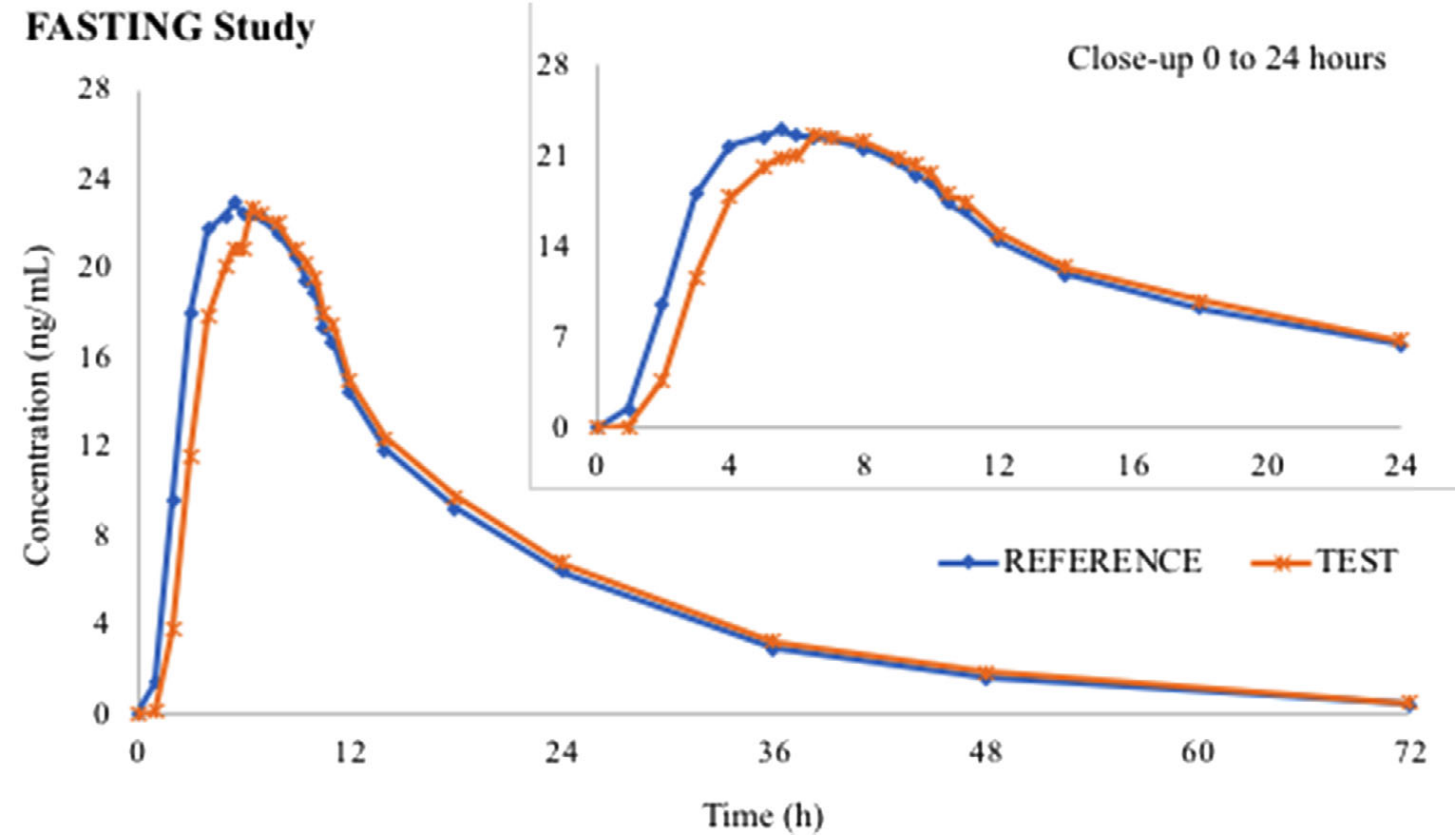
Duloxetine mean pharmacokinetic curves after Test and Reference administered as single dose in fasting state (N = 66).

**Figure 2:**
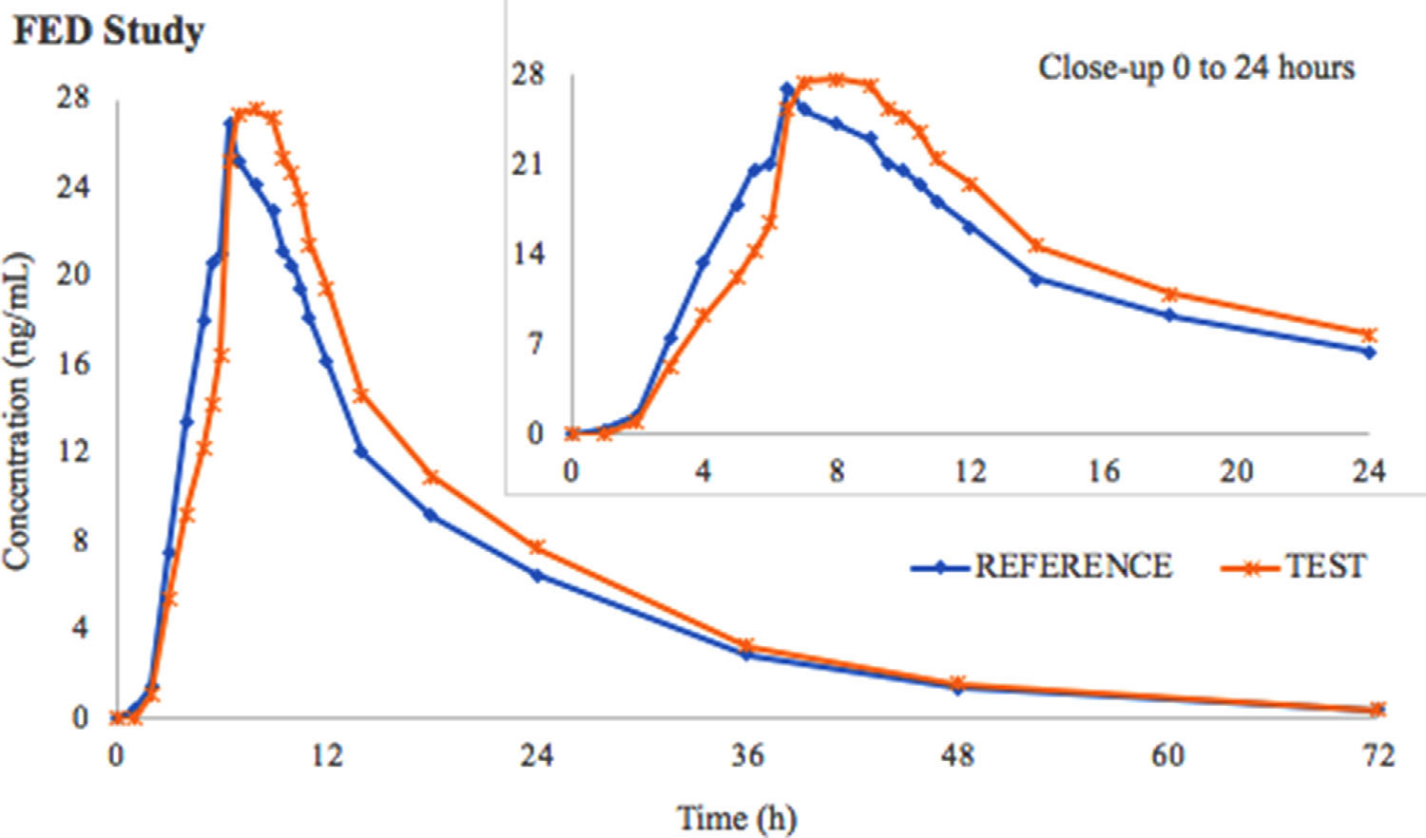
Duloxetine mean pharmacokinetic curves after Test and Reference administered as single dose under fed conditions (N = 42).

The mean pharmacokinetic parameters are summarized in Table 3, and mean duloxetine concentration‐time curves in fasting and fed states are shown in Figure 1 and Figure 2, respectively.

**Table 3:** Mean Pharmacokinetic Parameters of Duloxetine 60 mg Formulated as Gastroresistant Hard Capsules (Test or Reference), Administered as Single Dose to Fasting or Fed Healthy Volunteers

		C_max_ (ng/mL)	AUC_0‐t_ (ng/mL^★^h)	AUC_0‐∞_ (ng/mL^★^h)	T_max_ (h)	t*_½_* (h)
Study	Treatment	Mean (SD)	Mean (SD)	Mean (SD)	Median (Range)	Mean (SD)
Fasting study (N = 66)	Test	24.990	424.291	436.278	6.5	12.4
		(±15.756)	(±301.286)	(±316.751)	(3.0‐11.0)	(±2.8)
	Reference	26.542	426.554	438.139	5.0	12.0
		(±15.444)	(±302.663)	(±324.167)	(2.0‐9.0)	(±3.0)
Fed study (N = 42)	Test	32.820	442.302	449.568	7.0	10.5
		(±17.624)	(±263.342)	(±268.666)	(3.0‐11.0)	(±1.9)
	Reference	29.691	401.324	409.310	6.5	10.8
		(±17.155)	(±268.048)	(±275.808)	(1.0‐9.0)	(±2.3)

AUC indicates area under the plasma concentration–time curve; C_max_, peak concentration; T_max_, time to C_max_; t_½,_ elimination half‐life.

The point estimates of duloxetine pharmacokinetic ln‐transformed parameters and the 90%CIs for the ratios of the population means, along with the intrasubject coefficients of variation registered are shown in Table 4.

**Table 4:** Duloxetine Point Estimates, 90%CIs, and ISCV for C_max_ and AUC_0‐t_

Study	PK Parameter	T/R Ratio (%)	90%CI	ISCV (%)
Fasting study (N = 66)	C_max_	92.63	86.68‐98.98	23.13
	AUC_0‐t_	99.04	93.25‐105.18	20.95
Fed study (N = 42)	C_max_	111.98	105.24‐119.16	17.07
	AUC_0‐t_	111.50	105.64‐117.69	14.78

AUC indicates area under the plasma concentration–time curve; C_max_, peak concentration; ISCV, intrasubject coefficient of variation; PK, pharmacokinetic; T/R, test/reference.

The statistical evaluation of pharmacokinetic data showed that the 2 duloxetine formulations were bioequivalent in both fasting and fed states as the Test/Reference ratios for the geometric means of the primary parameters (C_max_ and AUC_0‐t_), and their corresponding 2‐sided 90%CIs were contained within the predefined regulatory limits of 80.00% to 125.00%.

#### Effect of Food on Duloxetine Bioavailability

According to the pharmacokinetic results of the 2 studies (see Table 3), following administration of duloxetine in fed conditions, C_max_ was increased by up to 30% while the extent of absorption was not influenced significantly (AUC_0‐t_ in fed state within ±6% as compared with fasting conditions), and time to reach peak plasma concentration (T_max_) was increased by 20%. The average delay in reaching peak plasma levels in fed state was 1.15 hours in both formulations.

### Safety Results

Seventy‐one subjects were included in the per‐protocol safety population in the fasting study, and 44 subjects in the fed study. The adverse events experienced during the 2 studies were mild or moderate in intensity and transient, with complete recovery being concluded before the study exit examinations.

In the fasting study a total of 69 events were reported after administration of 138 duloxetine single doses (0.50 events per fasting single‐dose exposure), and 46% of the 71 subjects dosed at least once experienced at least 1 adverse event.

In the fed study a total of 20 events were reported after administration of 86 duloxetine single doses (0.23 events per fed single‐dose exposure), and 25% of the subjects dosed at least once experienced at least 1 adverse event.

The statistical analysis of adverse events by formulation (1 proportion test) carried out in each study confirmed that there is no statistically significant difference between Test and Reference with regard to incidence of adverse events or incidence of subjects having experienced adverse events in either the fasting or the fed studies.

Based on the average number of adverse events per single‐dose exposure and the incidence within the study population of subjects having experienced adverse events, it appears that duloxetine is better tolerated when administered after a meal as compared with administration during prolonged fasting (from at least 8 hours before dosing until 6 hours postdose).

A listing of all adverse events reported during the 2 studies and judged by the investigators as having a reasonable causal relationship with the study medication and their incidence within the study population is presented in Table 5.

**Table 5:** Incidence of Adverse Events Within the Study Population After Single‐Dose Duloxetine (60 mg) in Fasting State and Under Fed Conditions

SOC and Individual AEs	Incidence of AE Within the Study Population (N = 71) in Fasting State % (n)	Incidence of AE Within the Study Population (N = 44) in Fed Conditions % (n)
Gastrointestinal disorders	49.3% (35 subjects)	9.1% (4 subjects)
Nausea	23.9% (17 subjects)	6.8% (3 subjects)
Vomiting	7.0% (5 subjects)	2.3% (1 subject)
Dry mouth	1.4% (1 subject)	…
Diarrhea	7.0% (5 subjects)	…
Epigastric pain	5.6% (4 subjects)	…
Constipation	1.4% (1 subject)	…
Abdominal pain	2.8% (2 subjects)	…
Pyrosis	…	2.3% (1 subject)
General disorders and administration site conditions	…	2.3% (1 subject)
Malaise	…	2.3% (1 subject)
Ear and labyrinth disorders	5.6% (4 subjects)	…
Vertigo	5.6% (4 subjects)	…
Investigations	…	2.3% (1 subject)
Blood pressure increased	…	2.3% (1 subject)
Nervous system disorders	26.8% (19 subjects)	6.8% (3 subjects)
Headache	21.1% (15 subjects)	2.3% (1 subject)
Dizziness	5.6% (4 subjects)	…
Paresthesia, distal	…	2.3% (1 subject)
Extremities burning sensation	…	2.3% (1 subject)
Musculoskeletal and connective tissue disorders	…	4.5% (2 subjects)
Dorsal pain	…	4.5% (2 subjects)
Skin and subcutaneous tissue disorders	4.2% (3 subjects)	…
Sweating	4.2% (3 subjects)	…

AE indicates adverse events; SOC, *Medical Dictionary for Regulatory Activities* System Organ Classes.

The incidence of adverse events was notably higher in the fasting group for the *Medical Dictionary for Regulatory Activities* System Organ Classes (SOC) gastrointestinal disorders and nervous system disorders. The incidence of gastrointestinal disorders within the treated study population was 49.3% in the fasting state versus 9.1% under fed conditions. Of the individual adverse events reported that pertained to this SOC, there was a notably higher incidence of nausea in the fasted state. The incidence of nervous system disorders within the treated study population was 26.8% in the fasting state versus 6.8% in fed conditions. Of the individual adverse events reported that pertained to this SOC, there was a notably higher incidence of headaches in the fasted state. The limitations of these observations derive from the fact that the 2 phase 1 studies conducted were not placebo controlled, and therefore, it cannot be definitively concluded that duloxetine itself exhibits better tolerance in fed state because a correlation between nausea/headache and prolonged fasting was not excluded as a confounding factor.

## Discussion

Two pivotal single dose studies, 1 in fasting and the other in fed conditions, were conducted with the purpose of investigating the bioequivalence between a generic 60‐mg duloxetine gastroresistant hard capsule formulation (manufactured by Medochemie Ltd, Cyprus) and the innovator product Cymbalta 60‐mg gastroresistant hard capsules (manufactured by Eli Lilly Netherlands BV (Utrecht, the Netherlands). The statistical evaluation of pharmacokinetic data showed that the 2 formulations are bioequivalent in both the fasting and fed states, as the Test‐Reference ratios for the geometric means of C_max_ and AUC_0‐t_ and their corresponding 2‐sided 90%CIs were contained within the predefined regulatory limits of 80% to 125%. Both studies have shown that, when administered as single dose in the same prandial state, the Test and Reference duloxetine treatments exhibit similar safety profiles.

The pharmacokinetic data (mean fed and fasting pharmacokinetic profiles) and safety data (adverse events) from these 2 bioequivalence studies were compared in order to evaluate the effect of food on the oral bioavailability and tolerability of duloxetine. The comparisons showed that administration of duloxetine under fed conditions increased C_max_ by up to 30% while having an insignificant effect on extent of absorption (AUC_0‐t_ in fed state within ±6% as compared with fasting conditions). Administration in fed conditions also increased mean T_max_. The average delay in reaching peak plasma levels in the fed state was 1.15 hours in both formulations. Even though peak plasma levels were substantially higher in the fed state, there was no negative impact on the drug's safety profile. The negligible variation in overall systemic exposure suggests that efficacy remains unchanged irrespective of administration conditions.

Judging by the average number of adverse events per single‐dose exposure (0.50 in fasting versus 0.23 in fed conditions) and the incidence of subjects having experienced adverse events within the study population (46% of the treated subjects in fasting conditions versus 25% of the treated subjects in fed conditions), duloxetine was better tolerated when administered after a meal as compared with administration during prolonged fasting. In particular, higher incidences of nausea and headache were noted in the fasting group. In the absence of a placebo group, prolonged fasting itself could not be excluded as a contributing factor that could have hypothetically increased the likelihood for gastric adverse events and headache. However, knowing the individual contribution of each factor within the observed food‐formulation safety interaction is, from a clinical perspective, less important than the global finding that duloxetine is better tolerated if taken with food.

The results of the bioavailability comparison are in accordance with the summary pharmacokinetic information made public by the innovator company, administration with food having indeed delayed the time to reach peak plasma concentrations while influencing the extent of absorption marginally. In addition, the current comparison showed that at a 60‐mg dose, food also increases peak duloxetine concentrations. With regard to tolerability, the results of our comparison are in accordance with other published data (the same improved tolerability of duloxetine 60‐mg dose when taken with food was concluded in a study done by Whitmyer et al^11^).

## Conclusions

In contrast to administration of duloxetine in the fasting state, administration of the drug after a high‐fat, high‐calorie breakfast increased C_max_ by up to 30% and delayed T_max_ by an average of 1.15 hours, although the extent of absorption (AUC_0‐t_) was insignificantly altered. Even though the peak plasma levels were substantially higher in the fed state, food increased duloxetine's tolerability. The negligible variation in overall systemic exposure suggests that efficacy remains unchanged irrespective of administration conditions; however, a better tolerability is expected for the 60‐mg dose if taken with food.
